# COVID-19-Related Health Literacy of Socioeconomically Vulnerable Migrant Groups

**DOI:** 10.3389/ijph.2022.1604664

**Published:** 2022-06-15

**Authors:** Didier Ruedin, Johanna Probst, Philippe Wanner, Denise Efionayi-Mäder, Patrick Bodenmann

**Affiliations:** ^1^ Swiss Forum for Migration and Population Studies, Université de Neuchâtel, Neuchâtel, Switzerland; ^2^ African Centre for Migration & Society, University of the Witwatersrand, Johannesburg, South Africa; ^3^ Institut de Démographie et Socioéconomie, Université de Genève, Geneva, Switzerland; ^4^ Department of Vulnerabilities and Social Medicine, University Center for General Medicine and Public Health, Lausanne, Switzerland

**Keywords:** health literacy, vulnerability, COVID-19, migration, information

## Abstract

**Objective:** Understand the COVID-19-related health literacy of socioeconomically vulnerable migrant groups.

**Methods:** We conducted a survey available in 8 languages among 2,354 members of the target population in Switzerland in 2020. We measured health literacy in four dimensions (finding, understanding, evaluating and applying health information) and assessed adherence to official recommendations during the COVID-19 pandemic.

**Results:** Most migrants felt well informed about the pandemic. Using an extended index of health literacy, we found a moderate correlation (*r* = −0.28 [−0.24, −0.32]) between COVID-19-related health literacy and socioeconomic vulnerability. The most socioeconomically vulnerable migrants tended to have more difficulty finding and understanding health information about COVID-19 and adhered more to unscientific theses that were not part of the official communication.

**Conclusion:** Special communication efforts by public health authorities have reached most migrants, but socioeconomic vulnerability can be a barrier to taking precautions.

## Introduction

When COVID-19 began to affect countries across the world in early 2020 and a pandemic was declared, it became clear that the entire population needed to be successfully reached for public health interventions. From the outset, some researchers voiced concerns that vulnerable sections of society would not be reached and would remain exposed to health risks associated with the pandemic [1–3], including migrants who do not speak the local language (well) and who are not familiar with the local health care system [4, 5]. These concerns revolved around health literacy, especially not understanding key messages in preventive measures (information) and drawing the right conclusions (behavior). Half a year into the pandemic, we evaluated the health literacy of potentially vulnerable migrants in Switzerland with a survey of 2,354 respondents and a targeted sampling strategy. We defined potentially vulnerable migrants as those who are members of migrant groups with a high share of socioeconomically vulnerable individuals, and we used a multidimensional measure of socioeconomic vulnerability to identify affected individuals.

We found that potentially vulnerable migrants generally feel well informed—on par with the general population [6] — but we found a subgroup of socioeconomically vulnerable migrants with lower health literacy. We identified migration-related vulnerability related to the stability of the residence status as a central element of vulnerability, an aspect commonly ignored in the literature. While we did not find that socioeconomically vulnerable migrants were considerably more exposed to the pandemic in consideration of the preventive measures taken, we nonetheless express concern about socioeconomic vulnerability regarding implementation as the pandemic continued, preventive measures were adapted and vaccination programs were rolled out. To help overcome vulnerabilities in implementation, successful interventions should involve official campaigns but also actively reach out to migrant communities, migrant media, and cultural and religious communities that are characterized by relatively high trust among the subgroup of socioeconomically vulnerable migrants.

Health literacy plays a central role in public health because it influences whether individuals make appropriate health decisions [7]. We adopted an understanding of health literacy as a multidimensional concept that involves access to relevant information, understanding of this information, and appraising the information to apply it in making informed health decisions [8]. In short, health literacy refers to the capacity of individuals to make decisions in their daily lives which have a positive influence on their health [7]. Many studies have examined differences in health literacy between various sections of society or the impact of health literacy on particular health decisions or health outcomes [8–11]. Indeed, many public health campaigns revolve around health literacy [12, 13].

While health literacy is often applied in general terms—the ability to understand and use health care information overall or across a wide range of situations—it equally applies to specific situations [8, 9]. Here, we applied the concept of health literacy to the COVID-19 pandemic: understanding and acting on information regarding the novel coronavirus (SARS-CoV-2). The pandemic is a special case, since the public has been exposed to such a large amount of information that the pandemic was referred to as an infodemic [9]. Some of this information was contradictory, some of it evolved over time, some of it was uncertain, and some of it was manifestly false, making health literacy no less important than is ordinarily the case [1, 9].

In such a context, a broad understanding of health literacy that includes the ability to appraise and act on information plays an important role in coping with the pandemic [1, 9]. Migrant populations may be affected because they often have limited language skills and may not be familiar with the local health care system [4, 14, 15]. Migrant populations may also be at risk because of socioeconomic vulnerability: many migrants work in occupations with low pay and are exposed to occupational hazards [3, 5]. It would be wrong to assume that all migrants are vulnerable, however, since there are also highly skilled migrants—particularly in Switzerland [16].

Socioeconomic vulnerability should be understood as a multidimensional concept, wherein different factors contribute to vulnerability [3, 17, 18]. It refers to life situations that are characterized by a combination of a low level of formal education, no or limited employment, limited financial resources, a precarious (or nonexistent) residence status, and an imperfect command of the local language. Vulnerability implies a greater risk of exposure to illness, but this risk needs to be understood in probabilistic rather than deterministic terms [18].

Thinking about socioeconomically vulnerable migrants and health literacy during the COVID-19 pandemic, it is not entirely clear whether we should expect a negative association between socioeconomic vulnerability and poor health literacy because information relevant to the pandemic may also be available from the migrants’ countries of origin [5]. Despite this, we expected the dominant association to be between greater socioeconomic vulnerability and lower health literacy, especially since health literacy also involves appraising and acting on information, and these two factors are inherently linked to the context of the country of residence.

## Methods

The outcome variable measured health literacy. We followed the definition and method proposed by Sørensen et al. [8] and measured four types of competencies—accessing, understanding, appraising, and applying health-related information—using 4-point answer scales. We used the questions from Orkan et al. [9], who applied these four types of competencies to COVID-19; they were adapted to the Swiss context by Vogt et al. [6]. We added a general assessment (“Generally speaking, how well do you feel informed about the coronavirus and the pandemic?”) because not all respondents may weigh different sources and competencies equally, and we added a series of factual questions. With the factual items, we did not rely entirely on subjective assessments. The factual items were coded −1 if incorrect, 0 if the response was “do not know,” and +1 if correct. The full questions are given in Supplementary SA1. The three components—self-declared health literacy, feeling informed, and factual questions—were standardized on a scale 0 to 1 and equally weighted in an index of health literacy related to COVID-19.

As a predictor, we used a multidimensional index of socioeconomic vulnerability [19, 20] combining 5 variables: local language skills, educational level, employment status, income level, and residence status. The index combines economic precarity and being at the margins of society and thus includes migration-related factors. Local language skills were assessed based on whether the respondents spoke one of the official Swiss languages, to what extent they could understand the local language, and whether they found filling in official forms difficult [21]. For employment status, we differentiated three cases: individuals with greater stability (employed, retired), with less stability (self-employed, students), and with the least stability (unemployed, on invalidity benefits). Income level considered household income and the response to a question of whether respondents could afford unexpected but necessary expenditures of various amounts. Residence status was coded to capture the stability of the status of the respondents, including irregular migrants without formal residence rights. The full questions and coding decisions are provided in Supplementary SA2. All five dimensions were standardized and equally weighted in the index of socioeconomic vulnerability. In addition, as illustrated in Supplementary SA3, we defined individuals with values over 0.6 on the index of socioeconomic vulnerability as particularly “vulnerable” to aid the presentation of results. None of the results presented substantively relied on this arbitrary cutoff.

The basis of our analysis was a representative sample drawn from register data. To identify potentially vulnerable migrants, we selected a random sample of foreign citizens born abroad from the sampling frame (SPH) of the Federal Statistical Office. We included persons aged 18 or older who were residents or asylum seekers, except short-term permit holders, who were born in a country corresponding to the survey languages (N = 1,669). However, we know from research on survey methodology that potentially vulnerable migrants are likely to be underrepresented in such a sample [22], so we complemented the sample with targeted recruitment through NGOs that work as multipliers (migrant media, support organizations, language schools) and work with socioeconomically vulnerable migrants (N = 685). With this approach, we sought a diverse sample of potentially vulnerable migrants in Switzerland. The descriptive summaries in Supplementary SA4 demonstrate that this approach worked well. With targeted sampling through NGOs, we recruited a higher share of vulnerable individuals: the sample was younger; less educated; had lower language skills; and included more unemployed individuals, refugees, asylum seekers, and irregular migrants who were more likely to struggle with unexpected expenses. We analyzed all cases jointly since the reported associations were substantially the same regardless of recruitment strategy (N = 2,354). The sample was balanced by sex, the median age was 37 years, the median residence in Switzerland was 7 years, and 12% were unemployed. Of the respondents, approximately 40% held a settled residence status, while 10% were provisionally admitted foreigners or irregular migrants, and the remainder had an annual residence permit or a short-term permit. Overall, varying degrees of socioeconomic vulnerability were represented in the sample.

The questionnaire essentially replicated Vogt et al. [6] and Orkan et al. [9]. While Orkan et al. [6] studied Germany, Vogt et al. [6] examined the general population in Switzerland in May 2020, allowing us to compare descriptive results with the general population on different aspects of health literacy. We modified some questions to adapt them to a migrant population and made the questionnaire available in eight languages (Albanian, Arabic, English, French, German, Portuguese, Spanish (only available in the NGO sample), and Tigrinya). The choice of languages, and thus the groups sampled, was inspired by the Swiss migrant population health monitoring program [23]. The survey was run entirely online, with invitations sent by conventional mail in the case of the random sample and through face-to-face contacts in the case of the NGO sample. The respondents in the NGO sample were incentivized with shopping vouchers of CHF20 that were distributed at the discretion of the organizations, and several NGOs offered linguistic support to the respondents. The fieldwork was undertaken between October and December 2020.

We complemented descriptive statistics with Bayesian regression models. The regression models were equivalent to OLS and used the uninformative default priors in the R package rstanarm [24], which regularizes the posterior. The default priors draw on the distribution of the observed values in the data and do not introduce any subjective biases—they are indicated in the caption notes of each model. In the central model, we used the index of COVID-19-related health literacy as the outcome variable and the index of socioeconomic vulnerability as the predictor. We report the median of the posterior as coefficients and the median absolute deviation (MAD) as robust measures of uncertainty equivalent to standard deviations. As control variables, we used sex, age, and whether a person had completed any kind of education in health care.

## Results

**FIGURE 1:**
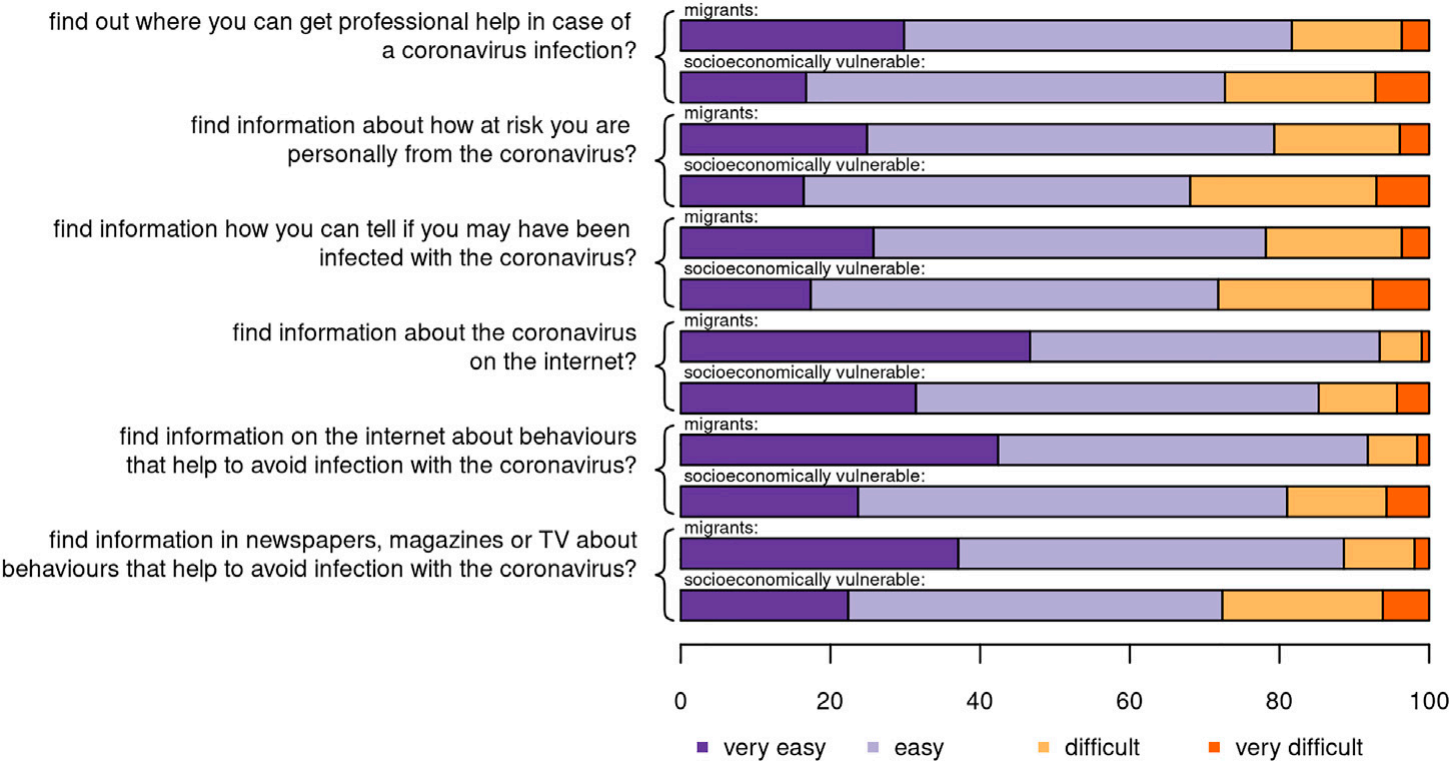
Finding COVID-19 related health information, potentially vulnerable migrants, Switzerland, November 2020. N = 2,354; socioeconomic vulnerable migrants with an index of 0.6 or higher. Questions are sorted by the sum of “easy” and “very easy” across both subsamples.

Looking at health-related information access, understanding, evaluation, and application, we found that potentially vulnerable migrants were relatively well informed. Compared to the results of a study asking the same questions in the general population [6], self-reported health literacy for the potentially vulnerable migrants in our study was equivalent. For example, Figure 1 shows the results for finding COVID-19-related health information. The figure also shows that health literacy was lower for the subsample of socioeconomically vulnerable migrants. For each question, socioeconomically vulnerable migrants reported more difficulty—as shown by the longer orange and red bars standing for “difficult” and “very difficult”.

Supplementary SA5 includes equivalent figures for the other dimensions of self-reported health literacy: understanding, evaluation, and application of COVID-19-related health information. In each case, we systematically found lower levels of health literacy for the socioeconomically vulnerable migrants. Of the different dimensions, evaluating COVID-19-related health information posed the most problems (especially the evaluation of information in the media). In addition to presenting these differences in graphical form, we calculated the mean score for each of the dimensions, finding lower levels of health literacy for all dimensions and all constituent items (average difference 0.2 on a 4-point scale, Supplementary SA5).

When asked how well informed they felt about the coronavirus and the pandemic, the potentially vulnerable migrants generally responded positively: 31.4 percent felt very well informed, and another 55.3 percent felt well informed. Compared to what Vogt et al. [6] reported for the general population in May 2020, the share of those participants who felt not so well informed (11.0%) was slightly higher, while there was no substantive difference for those not at all informed (2.3%). Looking at the subgroup of socioeconomically vulnerable migrants, the share of those very well informed was lower (25.9%), while the share of those not so well informed (16.7%) and not at all informed (4.6%) was noticeably higher.

**FIGURE 2:**
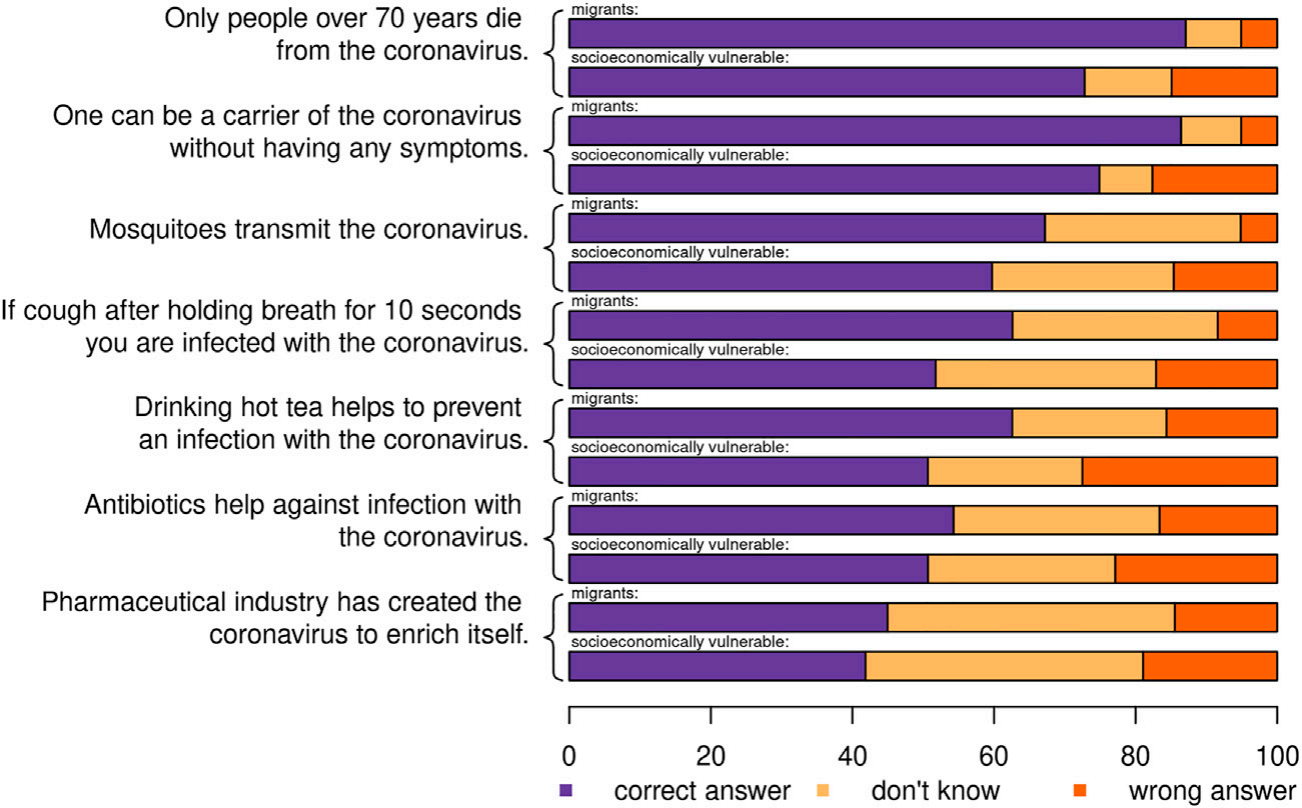
Factual questions on COVID-19, potentially vulnerable migrants, Switzerland, November 2020. Correct statement: carrier without symptoms; for the other (incorrect) statements, identifying them as “false” is coded as “correct answer”. N = 2,354; socioeconomic vulnerable migrants with an index of 0.6 or higher. Questions are sorted by the share of correct answers across both subsamples.

The factual questions in Figure 2 illustrate that self-assessed health literacy can be insufficient. While some statements were correctly classified by a majority of respondents, for others—like whether drinking hot tea helps to prevent an infection—we found more incorrect answers and a great deal of uncertainty. We have no comparable data for the general population, but we once again found a substantial difference for the subgroup of socioeconomically vulnerable migrants: On average, members of the subgroup of socioeconomically vulnerable migrants correctly answered 2.7 factual questions, compared to 3.1 correct answers for the wider group of potentially vulnerable migrants.

**FIGURE 3:**
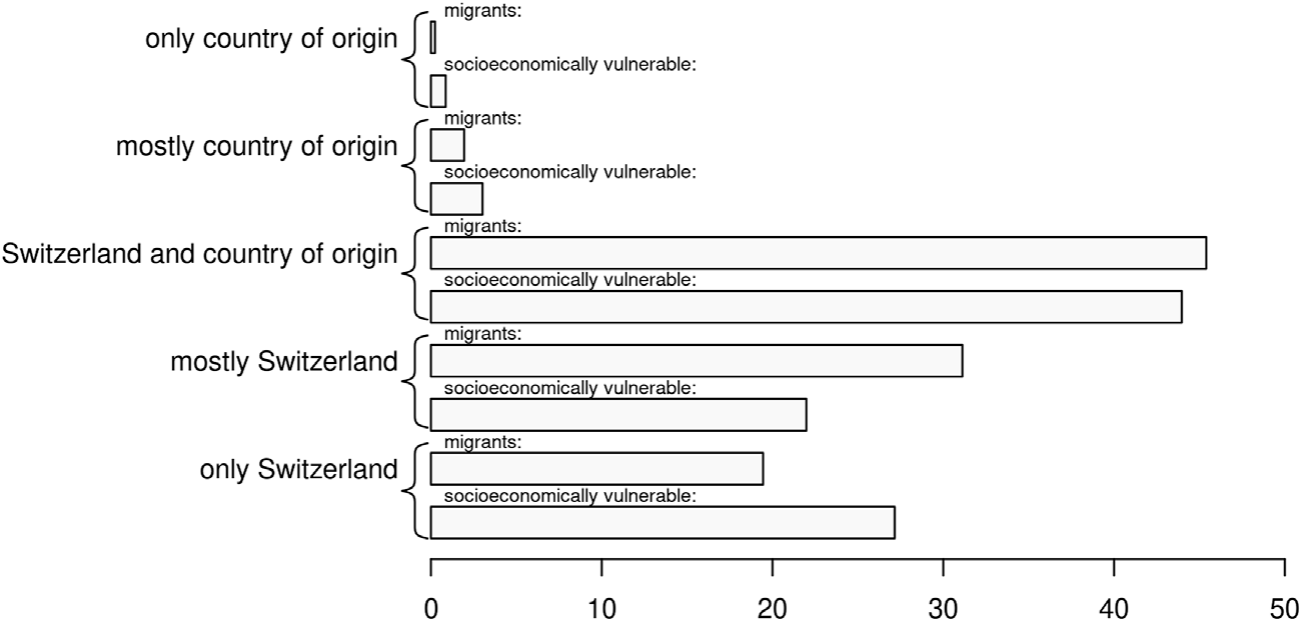
Source of the information on COVID-19 consulted by potentially vulnerable migrants, Switzerland, November 2020. N = 2,354; socioeconomic vulnerable migrants with an index of 0.6 or higher.

For migrants, especially for those with limited language skills in the country of residence, the origin of the information may influence health literacy. Figure 3 shows that most of the potentially vulnerable migrants relied either entirely or mostly on information from Switzerland or on a mix of sources. Very few of the potentially vulnerable migrants relied exclusively on information from their countries of origin. For the subgroup of socioeconomically vulnerable migrants, we found a higher share relying entirely on information from Switzerland (27.2%) but also a higher share relying on information from the country of origin (0.9% entirely, 3.0% mostly, with little difference for those mixing sources equally).

In Supplementary SA6, we present a list of sources used by potentially vulnerable migrants, noting that the distribution of sources corresponds largely to what Vogt et al. [6] reported for the general population. Socioeconomically vulnerable migrants, however, differed to some extent from this pattern. Overall, socioeconomically vulnerable migrants used fewer sources (3.9 sources on average, compared to 4.9 sources for potentially vulnerable migrants). In comparison, socioeconomically vulnerable migrants were more likely to use social media as a source of health information, as well as people within the cultural or religious community. Noticeably lower were the use of television, newspapers, radio, and health authorities.

Looking at the trust in the different sources of information in Supplementary SA7, we found high levels of trust in health authorities, health experts, and the official information posters (all above 80% for trusting “rather” or “very much”). In terms of trust, there were no substantive differences between different news media—television, radio, newspapers—and internet websites. The least trusted source was social media. This distribution largely holds for the subgroup of socioeconomically vulnerable migrants, but there were important differences: The share of respondents trusting social media was approximately 10 percentage points higher for socioeconomically vulnerable migrants, while the share of migrants trusting migrant media and people within the cultural or religious community was approximately 20 percentage points higher. The share of socioeconomically vulnerable migrants trusting migrant media “very much” was substantially larger. Supplementary SA8 shows high compliance with the official measures, but socioeconomically vulnerable migrants reported slightly fewer measures taken.

**FIGURE 4:**
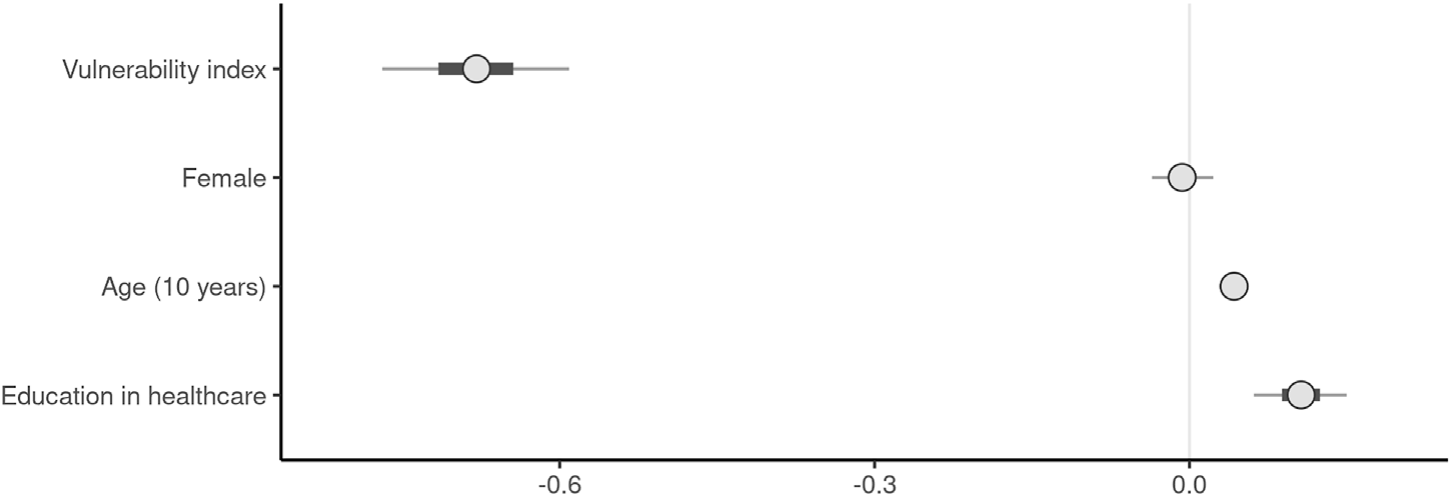
Regression model for health literacy, Switzerland, November 2020. Shown are the coefficients of the regression model in graphical form. Outcome variable: health literacy index, N = 2301. Median of the posterior as coefficients (circles), median absolute deviation (MAD) as robust standard deviations as lines. Circles to the left of the zero line indicate negative coefficients (e.g., higher value on vulnerability index = less health literacy); circles to the right of the zero line positive coefficients (e.g., education in healthcare = more health literacy). See Supplementary SA9 for full table.

Overall, there was a moderate negative correlation between health literacy and socioeconomic vulnerability (r = −0.28 [95% CI −0.24, −0.32]). To identify who was more likely to exhibit high levels of health literacy, we used regression analysis. The model presented graphically in Figure 4 shows that the socioeconomically vulnerable migrants had considerably lower levels of health literacy than the other potentially vulnerable migrants. The circle representing the coefficient for the index of vulnerability is clearly left of the dotted zero line, indicating a substantially important association between greater socioeconomic vulnerability and lower health literacy.

To better understand the different facets of the multidimensional vulnerability index, we also ran this regression model with each constituent separately (Supplementary SA9). Since these constituents were standardized to values between 0 and 1 and the data were the same, we could directly compare the regression coefficients, although qualitatively they captured different aspects of socioeconomic vulnerability among the migrants. The largest coefficient was for residence status (0.35), followed by education (0.32), language (0.18), employment status (0.15), and income (0.08). All these constituents yielded substantive associations with health literacy. With the stability of the residence status, we highlight a factor neglected in the literature. The models showed that socioeconomically vulnerable migrants were affected not only by difficulties in language and communication. The relatively small coefficient for language in this case may have been influenced by the availability of COVID-19-related information in multiple languages as well as access to material from the country of origin.

In a separate step, we used regression models to predict individual exposure to COVID-19 through not taking recommended preventive measures. We focused on measures that most individuals should be able to take—avoiding handshakes, washing hands, sneezing in one’s elbow, keeping distance, wearing masks, and wearing masks on public transport—but we found that socioeconomically vulnerable migrants were more exposed to COVID-19 because they were less likely to take the recommended precautions. If we talk about exposure, we need to bear in mind the already greater exposure from aspects that the migrants cannot control individually, such as living in smaller housing spaces [25], being in high exposure jobs [26], or not being entitled to work from home [27]. With the data at hand, we cannot enumerate these more consequential aspects of exposure. Less exposure occurred for women, older individuals, and those with education in health care (Supplementary SA9). The reported difference was substantially small (coefficient 0.34 on a scale from 0 to 6). Where there were clear differences in the uptake of measures for the subgroup of socioeconomically vulnerable migrants (namely, (self-) quarantine in case of symptoms or after contact with a person who has tested positive), this difference mostly occurred because the measure did not apply to the respondents or because it was “not possible” for them to take such measures. Comparing the migrants’ compliance with measures against COVID-19 with that of the general population in Vogt et al. [6], we identified great similarity between the potentially vulnerable migrant population and the general population. While socioeconomically vulnerable migrants had lower health literacy than the general population, we have no clear evidence that this would lead to greater exposure to COVID-19 to the extent we can measure this with generic measures recommended against COVID-19, such as wearing masks.

## Discussion

The COVID-19 pandemic made it clear that health information and health literacy play a central role in public health. In the context of information overload, it was all the more important to ensure that all sections of society were able to access and understand essential health information, evaluate its reliability and apply it in their daily lives. Early in the pandemic, concerns were raised about vulnerable migrants potentially being left out of official campaigns [1], but given the global reach of the pandemic, migrants were probably able to obtain information from their countries of origin if desired or needed. We found that the health literacy of potentially vulnerable migrants was comparable to what Vogt et al. [6] reported for the general population of Switzerland, so it would be wrong to associate migrants with poor health literacy in general [for a similar finding in Finland, see [28]]. At the same time, we demonstrated that socioeconomic vulnerability is associated with lower health literacy among the migrant population.

We showed that different components of socioeconomic vulnerability have independent effects on health literacy, indicating that a broad and multidimensional approach to measuring vulnerability is warranted to better understand public health. In particular, we showed that residence status (or lack thereof in the case of irregular migrants) is a form of vulnerability that is negatively associated with health literacy. This demonstrates that nationality or being born abroad is the wrong unit of analysis with regard to health literacy and vulnerability. Indeed, we sampled potentially vulnerable migrants by nationality, as in previous studies (e.g., [23]), and found that many of them were well informed about the COVID-19 pandemic—with an important subgroup of socioeconomically vulnerable migrants that could be identified by using a multidimensional approach to vulnerability. Another important dimension concerns material circumstances, which may in particular affect the competency of acting on health information, such as when working from home is impossible for many migrant workers, or quarantine is associated with a loss of income for workers paid by the hour [27]. While information certainly plays a key role in combating the pandemic, material aspects should not be neglected.

On a methodological note, we started with a sample of potentially vulnerable migrants using the population register and nationality as the basis. We found that targeted sampling via NGOs is a good complement to the register-based sample. Perhaps the targeted sample can be considered preferable for studies with a clear focus on the subgroup of socioeconomically vulnerable migrants—a subgroup that cannot be identified directly in the register data. Put differently, without targeted sampling, the proportion of well-informed migrants in the sample may be comparatively high. Especially in studies working with a more modest sample size, the subgroup of socioeconomically vulnerable migrants may be missed [22], and unless the sample size is very large, general population surveys will miss them altogether. In the present study, recruitment via NGOs and incentives for respondents worked well.

Socioeconomic vulnerability was associated with using fewer information sources overall, but a relatively higher share of respondents relied on social media and migrant media—although social media was not well trusted. With that, we found that socioeconomically vulnerable migrants were more exposed to “fake news” [see also [29, 30]], which can be problematic in a context of information overload, especially as the pandemic developed and information from different countries may not have been reinforcing as countries developed different measures [31–33]. Our factual items identified a small but substantial minority of respondents holding erroneous views on COVID-19-related health questions, the share of which was higher among the subgroup of socioeconomically vulnerable migrants.

In addition to highlighting the plight of socioeconomically vulnerable migrants, we also identified ways to reach them through migrant media and cultural and religious organizations, which constitute relatively well trusted and important sources of information for this part of the population [30, 34]. At the same time, government information is also well trusted across the population, so targeted campaigns should not replace but rather complement public health campaigns. Certainly, this implies making relevant material available in different languages but then working with different migrant communities and NGOs to actively disseminate campaign messages—be this in the context of future pandemics, vaccination efforts, or for the dissemination of other health messages targeted at subgroups of migrants.

In conclusion, while migrants have the same average level of health literacy related to COVID-19 as the general population, we identify a subgroup of socioeconomically vulnerable migrants who exhibit lower levels of health literacy in all dimensions considered. Thus, special attention is needed when evaluating public health messages to ensure that the target population is reached. Besides translating key public health messages, active and specific communication efforts are required to reach socioeconomically vulnerable migrant communities.
