# Supplementary material for: COVID-19-Related Health Literacy of Socioeconomically Vulnerable Migrant Groups
Source: Int J Public Health. 2022 Jun 15;67:1604664. doi: 10.3389/ijph.2022.1604664 (PMC9240819; doi:10.3389/ijph.2022.1604664)
Supplement: Supplementary file 1 [file DataSheet1.PDF]

COVID-19 related health literacy of socioeconomically vulnerable migrant groups

Supplement

A1 Question Wording for Health Literacy

“It is not always easy to find out about issues related to the coronavirus and the pandemic and to draw the right conclusions. Please indicate how easy or difficult the points are for you personally.” [Very easy, Easy, Difficult, Very difficult]

Finding Information

find information about the coronavirus on the internet?

find information on the internet about behaviours that help to avoid infection with the coronavirus?

find information in newspapers, magazines or television about behaviours that help to avoid infection with the coronavirus?

find information how you can tell if you may have been infected with the coronavirus?

find out where you can get professional help in case of a coronavirus infection?

find information about how at risk you are personally from the coronavirus?

Understanding Information

understand the instructions of professionals (e.g. doctors, pharmacists, nurses) on protective measures against the coronavirus?

understand the coronavirus-related behaviour and hygiene instructions of the Federal Office of Public Health (FOPH)?

understand advice from family members or friends on protective measures against the coronavirus?

understand information in the media about how to protect yourself from infection with the coronavirus?

understand what you find on the internet about the risks of the coronavirus?

understand what you find in newspapers, magazines or television about the risks of the coronavirus?

Assess Information

assess whether the information in the media about the coronavirus and the pandemic is reliable?

assess which behaviours carry a particularly high risk of infection with the coronavirus?

assess what protective measures you should take against infection with the coronavirus?

assess how vulnerable you are personally to the coronavirus?

assess whether you may have been infected with the coronavirus?

Decide on Information

use information from the media to decide how to protect yourself from infection with the coronavirus?

follow the instructions of professionals (e.g. doctors, pharmacists, nurses) to protect yourself from infection with the coronavirus?

use information from professionals (e.g. doctors, pharmacists, nurses) to decide how to deal with a possible coronavirus infection and disease?

use the information provided by the Federal Office of Public Health (FOPH) and other authorities to decide how to deal with a possible coronavirus infection and illness?

use information from the media to decide how to deal with a possible coronavirus infection and illness?

behave in a way that you won't infect other people?

Factual Questions

"According to you, is the following information on the coronavirus correct or incorrect?" [Correct, Incorrect, Don't Know]

If you cough after holding your breath for 10 seconds it means that you are infected with the coronavirus.

Antibiotics help against infection with the coronavirus.

One can be a carrier of the coronavirus without having any symptoms.

Only people over 70 years die from the coronavirus.

Mosquitoes transmit the coronavirus.

The pharmaceutical industry has created the coronavirus to enrich itself.

Drinking hot tea helps to prevent an infection with the coronavirus.

Additional Subjective Assessment Question

"Generally speaking, how well do you feel informed about the coronavirus and the pandemic?" [Very well-informed, Well informed, Not so well-informed, Not at all well-informed]

A2 Question Wording and Coding Decisions for Vulnerability

Table 1 Question wording and coding decisions for socioeconomic vulnerability

Dimension	Question	Coding
Language	"What is your main language, i.e. the language in which you think and that you know best?" and "Which other language do you consider as your main language?"	1 = main language spoken (first or second answer) = German, French, Italian; 0 = otherwise
	"Please assess how well you understand the language spoken in your place of residence in Switzerland."	0 = badly; 0.25 = rather badly; 0.5 = average; 0.75 = rather well, 1 = well
	"How confident are you filling out administrative forms by yourself in the local language?" [taken from 1,validated in 2]	0 = not confident at all; 0.3 not confident; 0.7 confident; 1 = very confident
Education	"What is the highest educational level you have achieved?"	0 = compulsory school not completed; 0.2 = compulsory school completed; 0.4 = short training in a company (up to 1 year); 0.5 = longer training in a company (more than 1 year); 0.6 = vocational training; 0.8 = high school (general or academic diploma, technical college or equivalent); 1 = university degree
Employment status	"What is your employment status?"	0 = unemployed, invalidity benefits, other; 0.5 = self-employed, student, military; 1 = employed, retired,

		housewife/husband
Income	"What is your net household income per month?"	0.125 = less than CHF 2000; 0.25 = 2000-4000; 0.375 = 4000-6000; 0.5 = 6000-8000; 0.625 = 8000-10000; 0.75 = 10000-12000; 0.875 = 12000-14000, 1 = more than 14000
	"Can your household currently afford an unexpected but necessary expense of" [adapted from 3]	0.2 = CHF 50; 0.4 = CHF 100; 0.6 = CHF 300; 0.8 = CHF 1,000; 1 = CHF 5,000
Residence status	"What is your residency status?"	1 = Swiss citizenship; 0.8 = C permit; 0.7 = B permit; 0.5 = L permit; 0.3 = F permit; 0.2 = N permit; 0 = no residence status

The column "Coding" indicates how numerical values were assigned to the response categories. The language levels included a small explanation ("Badly. I hardly understand anything.", "Rather badly. I understand some words and simple, daily expressions, can read a poster or sign with little text.", "Average. I understand frequently used phrases on familiar topics or daily life situations (family, shopping, work).", "Rather well. I understand the overall content of texts, conversations or radio/TV broadcasts on different topics if they employ clear standard language.", "Well. I understand almost everything, can follow any conversation and read texts of all kind without difficulties."). All components of the language dimension are equally weighted.

A3 Identifying Socioeconomic Vulnerable Migrants

In the binary indicator, individuals with a value on the index of more than 0.6 are considered "socioeconomically vulnerable". This corresponds to 10.2 percent of the sample. This binary indicator is used in the figures and tables in the supplement; respondents below the cutoff are referred to as "potentially vulnerable".

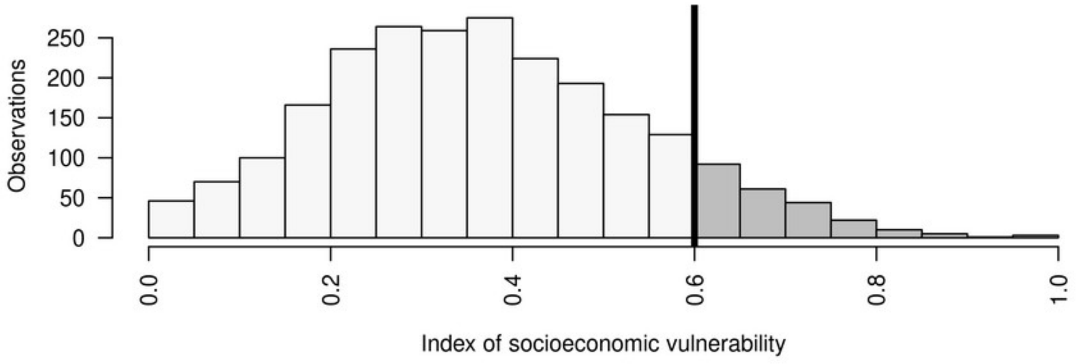

Figure 1 Distribution of the vulnerability index

For presentational reasons, the graphics in this article compare individuals above and below this cutoff.

A4 Sample Description

This table describes the sample of potentially vulnerable migrants.

Table 2 Sample description

Indicator	Sample (total)	NGO	Register-based
number of observations	2354	685	1669
% women	50.9	54.5	49.4
median age	37	33	38
% born abroad	98.7	96.3	99.6
% not complete primary school	9.8	13.6	8.3
% completed primary school	23.5	19.1	25.3
% completed university	28.9	31.8	27.7
% employed	42.0	23.3	49.5
% self-employed	8.2	9.9	7.5
% unemployed	11.5	15.4	9.9
% in education (student)	10.2	21.2	5.8
residence in Switzerland (median, years)	7	4	9
% Swiss national	2.7	7.4	0.7
% settled (permit C)	39.5	16.8	48.9
% resident (permit B)	43.5	43.4	43.6
% short-term (permit L)	0.6	0.1	0.8
% provisionally admitted (permit F)	9.0	17.2	5.7
% asylum seeker (permit N)	2.3	7.6	0.1
% irregular (*sans-papier*)	1.8	6.0	0.1
% language: understand “badly”	3.4	3.9	3.2

% understand “rather badly”	8.8	13.5	6.9
% understand “average”	28.2	35.5	25.2
% understand “rather well”	23.7	23.8	23.7
% understand “well”	34.1	21.1	39.4
% chronic illness (one)	17.0	18.3	16.5
% multiple chronic illnesses	6.6	8.5	5.9
% struggle with CHF 50 unexpected expenses	18.7	23.8	16.6

selected categories reported for education and professional status, 0.6% did not declare their residence status, 1.7% did not indicate language abilities, question wording for unexpected expenses: “Can your household currently afford an unexpected but necessary expense of” with maximal amount CHF 50. The status of being “born abroad” is self-reported.

A5 Understanding, Evaluating, and Applying Information

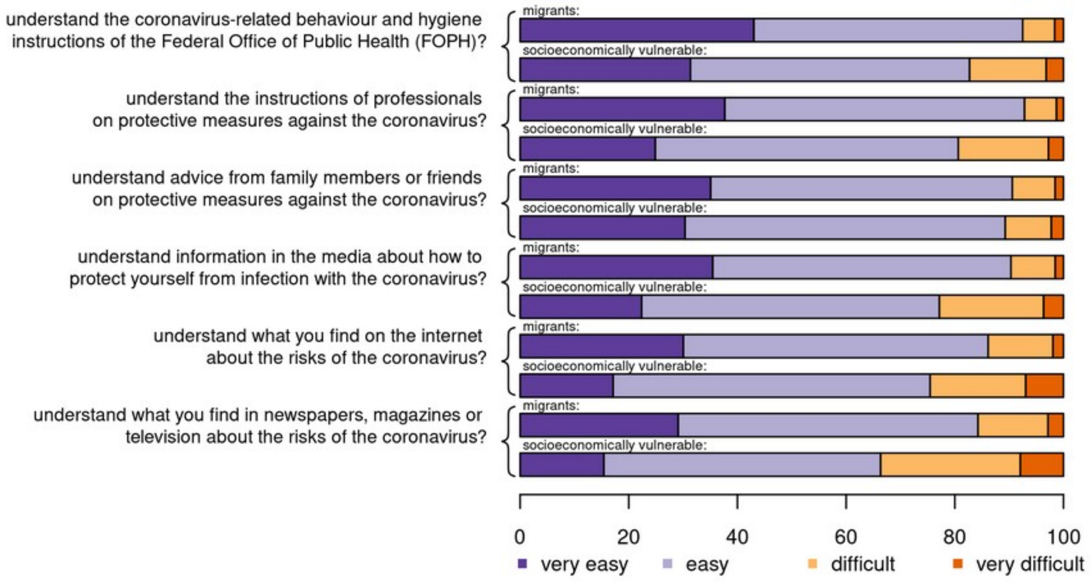

Figure 2 Understanding COVID-19 related health information, potentially vulnerable migrants, Switzerland, November 2020

N=2,354; socioeconomic vulnerable migrants with an index of 0.6 or higher. Questions are sorted by the sum of “easy” and “very easy” across both subsamples.

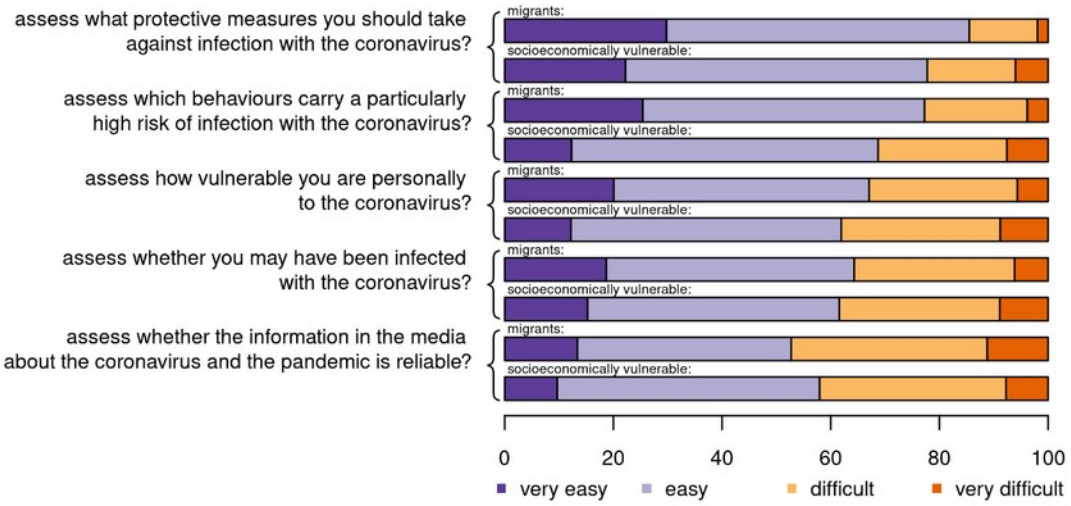

Figure 3 Evaluating COVID-19 related health information, potentially vulnerable migrants, Switzerland, November 2020

N=2,354; socioeconomic vulnerable migrants with an index of 0.6 or higher. Questions are sorted by the sum of “easy” and “very easy” across both subsamples.

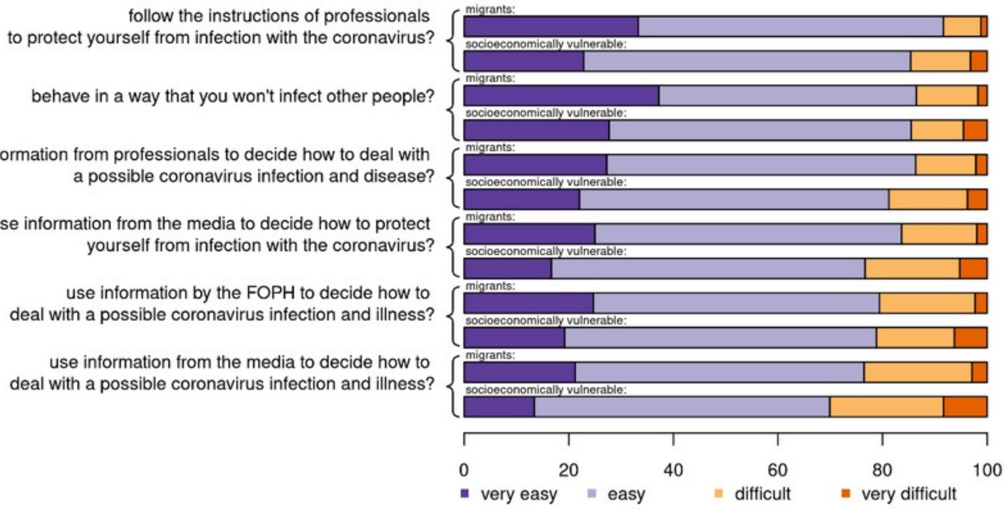

Figure 4 Applying COVID-19 related health information, potentially vulnerable migrants, Switzerland, November 2020

N=2,354; socioeconomic vulnerable migrants with an index of 0.6 or higher. Questions are sorted by the sum of “easy” and “very easy” across both subsamples.

Table 3 Mean score for different dimensions of health literacy, potentially vulnerable migrants, Switzerland, November 2020

Dimension	Potentially vulnerable migrants	Socioeconomic vulnerable migrants
Finding information	3.1	2.9
Understanding information	3.2	3.0
Evaluating information	2.8	2.7
Applying information	3.1	2.9

Answers “very easy” were coded as 4, “easy” as 3, “difficult” as 2, and “very difficult” as 1 so that higher values indicate greater health literacy. Given is the mean values for all items in a dimension; lower health literacy exists for all items of all dimensions. The biggest differences are for finding information about the coronavirus, finding information about how to avoid infection with the coronavirus on the internet, in newspapers or on television, and understanding information from newspapers, magazines, or television.

A6 Source of Information by Vulnerability

Internet usage in Switzerland is widespread across different parts of society. While 98% of the population with tertiary education used the internet in 2018, the share among the population with no more than primary education was still 80% [4]. Some NGO provided linguistic assistance to respondents in the targeted sample.

Table 4 Percentage of potentially vulnerable migrants using differences sources of information, Switzerland, November 2020

Source	Potentially vulnerable migrants	Socioeconomically vulnerable migrants
Television	61.4	49.0
Internet	60.8	45.6
Family, friends, family	50.5	45.6
Health authorities	51.5	34.4
Social media	45.5	46.9
Official posters of the FOPH	44.0	25.3
Online news	43.7	23.2
Doctor or health experts, e.g. pharmacist, nurse	29.1	22.8
Radio	29.3	12.0
News app	26.6	15.4
Printed newspaper	23.7	19.9
People of your cultural or religious community	11.3	22.0
Migrant media	10.6	13.7
Teletext	5.3	5.0
Telephone hotline	4.9	4.1

Multiple answers possible; N=2,354 individuals.

A7 Trust in Information

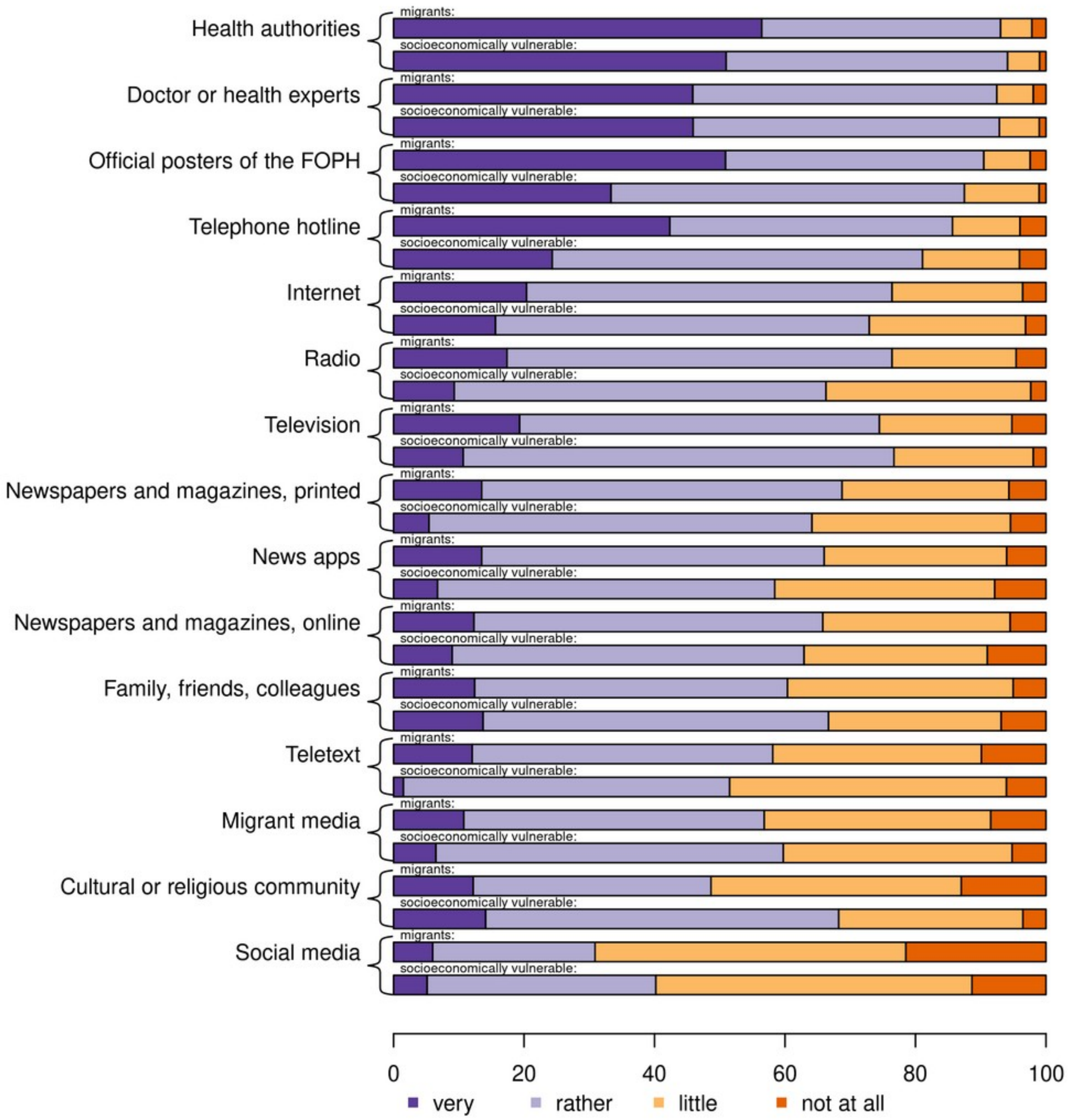

Figure 5 Trust in information sources by potentially vulnerable migrants, Switzerland, November 2020

Items are sorted by the sum of “little and “not at all” across both subsamples. N=2,354; socioeconomic vulnerable migrants with an index of 0.6 or higher.

A8 Measures Taken

The distribution of measures taken by the subgroup of socioeconomically vulnerable migrants is not substantively different for any of the items.

Figure 6 Measures taken against COVID-19 by potentially vulnerable migrants, Switzerland, November 2020

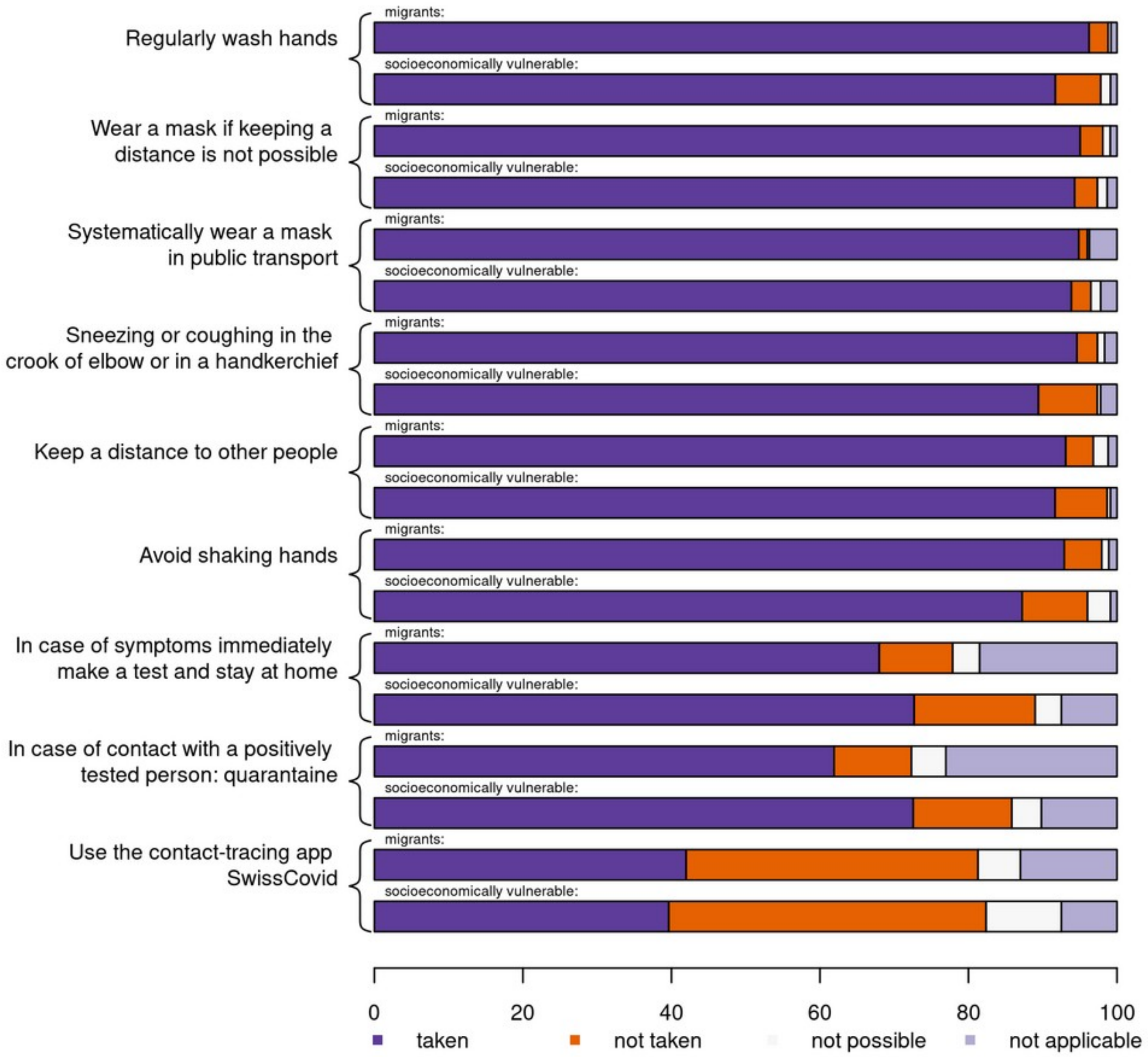

N=2,354; socioeconomic vulnerable migrants with an index of 0.6 or higher.

To calculate the number of measures not taken, we only considered those measures that can be followed by everyone: avoid handshakes, wash hands, not sneeze into hands, keep distance, wear masks in public, wear masks in public transport. We assigned a value of 0 if the measure was taken, a value of 0.5 if it was not possible, and a value of 1 if the measure was not taken. We then calculate the mean across these measures. Potentially vulnerable migrants have an average value of 0.21, and the subgroup of socioeconomi-

cally vulnerable migrants an average value of 0.33, indicating less adherence to these measures.

A9 Regression Models

Health Literacy and Vulnerability

Model M1 is presented graphically in Figure 4.

Table 5 Basic regression models for health literacy

Variable	M0	M1
Vulnerability index	-0.74 (0.05)	-0.68 (0.05)
Female		-0.01 (0.02)
Age (10 years)		0.04 (0.01)
Education in healthcare		0.11 (0.03)

Outcome variable: health literacy index, N=2301, default uninformative priors: Normal(0, 6.6) for the vulnerability index, Normal(0, 2.2) for female, Normal(0, 0.9) for age, and Normal(0, 3.2) for education in healthcare. Given are the median of the posterior as coefficients, and median absolute deviation (MAD) as robust standard deviations in brackets. All Rhat < 1.01.

Constituents of Vulnerability

Table 6 Extended regression models for health literacy

Variable	M3	M4	M5	M6	M7
Language index	0.18 (0.03)				
Education		0.32 (0.02)			
Employment status			0.15 (0.02)		
Income index				0.08 (0.03)	
Migration status					0.35 (0.05)
Female	-0.01 (0.02)	-0.01 (0.02)	-0.01 (0.02)	-0.02 (0.02)	-0.02 (0.02)
Age (10 years)	0.05 (0.01)	0.04 (0.01)	0.04 (0.01)	0.05 (0.01)	0.03 (0.01)
Education in healthcare	0.13 (0.03)	0.12 (0.03)	0.14 (0.03)	0.16 (0.03)	0.14 (0.03)

Outcome variable: health literacy index, N=2301, default uninformative priors: Normal(0, 3.8) for the language index, Normal(0, 3.0) for education, Normal(0, 2.6) for employment status, Normal(0, 3.4) for the income index, Normal(0, 5.9) for migration status, Normal(0, 2.2) for female, Normal(0, 0.9) for age, and Normal(0, 3.2) for education in healthcare. Given are the median of the posterior as coefficients, and median absolute deviation (MAD) as robust standard deviations in brackets. All Rhat < 1.01.

Exposure through Measures Not Taken

Table 7 Regression models for exposure through measures not taken

Variable	M8	M9
Vulnerability index	0.34 (0.08)	0.30 (0.09)
Female		-0.13 (0.03)
Age (10 years)		-0.07 (0.01)
Education in healthcare		-0.05 (0.04)

Outcome variable: exposure through measures not taken (sum of measures not taken, coded 1 if not taken, 0.5 if unable, and 0 if taken; shaking hands, washing hands, sneezing in elbow, keep distance, wearing masks, wearing masks in public transport). N=2301, default uninformative priors: Normal(0, 10.3) for the vulnerability index, Normal(0, 3.5) for female, Normal(0, 1.4) for age, and Normal(0, 5.1) for education in healthcare. Given are the median of the posterior as coefficients, and median absolute deviation (MAD) as robust standard deviations in brackets. Rhat < 1.01.

References

1. Chew LD, Griffin JM, Partin MR, Noorbaloochi S, Grill JP, Snyder A, et al. Validation of Screening Questions for Limited Health Literacy in a Large VA Outpatient Population. Journal of General Internal Medicine. 2008;23(5):561–6.

2. McNaughton C, Wallston KA, Rothman RL, Marcovitz DE, Storrow AB. Short, Subjective Measures of Numeracy and General Health Literacy in an Adult Emergency Department. Academic Emergency Medicine. 2011;18(11):1148–55.

3. Harder N, Figueroa L, Gillum RM, Hangartner D, Laitin DD, Hainmueller J. Multidimensional measure of immigrant integration. Proceedings of the National Academy of Sciences. 2018;201808793.

4. BFS. Internetnutzung in den Haushalten im Jahr 2019 [Internet]. Bern: Bundesamt für Statistik; 2019 [cited 2020 Mar 1]. Available from: https://www.bfs.admin.ch/bfsstatic/dam/assets/11127962/master
